# Observational Behavior Assessment for Psychological Competencies in Police Officers: A Proposed Methodology for Instrument Development

**DOI:** 10.3389/fpsyg.2021.589258

**Published:** 2021-02-26

**Authors:** Matthijs Koedijk, Peter G. Renden, Raôul R. D. Oudejans, Lisanne Kleygrewe, R. I. Vana Hutter

**Affiliations:** ^1^Department of Human Movement Sciences, Faculty of Behavioural and Movement Sciences, Vrije Universiteit Amsterdam, Amsterdam, Netherlands; ^2^Institute of Brain and Behavior Amsterdam, Amsterdam, Netherlands; ^3^Faculty of Health, Nutrition and Sport, The Hague University of Applied Sciences, The Hague, Netherlands; ^4^Faculty of Sports and Nutrition, Amsterdam University of Applied Sciences, Amsterdam, Netherlands

**Keywords:** psychological competence, assessment instrument, behavioral observation, psychological observation, professional performance, police

## Abstract

This paper proposes and showcases a methodology to develop an observational behavior assessment instrument to assess psychological competencies of police officers. We outline a step-by-step methodology for police organizations to measure and evaluate behavior in a meaningful way to assess these competencies. We illustrate the proposed methodology with a practical example. We posit that direct behavioral observation can be key in measuring the expression of psychological competence in practice, and that psychological competence in practice is what police organizations should care about. We hope this paper offers police organizations a methodology to perform scientifically informed observational behavior assessment of their police officers’ psychological competencies and inspires additional research efforts into this important area.

## Introduction

Psychological competence is important for the professional performance of police officers (e.g., Blum, 2000; Broomé, 2014; Andersen et al., 2015; van der Meulen et al., 2018). Psychological competence can be presumed to be an integration of multiple competencies (Fouad et al., 2009) and can be conceptualized as the knowledge, skills, and attitudes that police officers must possess to navigate the increasing psychological demands of their job. Psychological competence enables police officers to cope with stressful situations and make decisions and act under pressure (Nieuwenhuys and Oudejans, 2012, 2017). It would thus be important to test and train psychological competencies of police officers.

However, in current police practice, assessment of psychological competencies is rather reductionistic. For numerous reasons (including ease of administration and interpretation), psychological competence is picked apart into separate psychological constructs (pure constructs) that can be measured by questionnaires and self-reports. Although theoretically and methodologically grounded, a fundamental question with this approach is whether the assessment of pure constructs has predictive value for psychological competence as required in practice. In the end, it is the quality of behavior (i.e., decisions and skill execution) of officers that best shows psychological competence on the job, for instance, when having to perform under threatening and stressful circumstances. Yet, police organizations seem to consider measuring pure constructs as the most feasible and interpretable way of testing psychological competence (Baumeister et al., 2007; Marshall et al., 2020). This means that police organizations use techniques that are based on the reduction of personal characteristics to one specific construct, such as emotional resilience (e.g., Cochrane et al., 2003; Varela et al., 2004).

Moreover, in picking competence apart in separate constructs, the main focus has been on personality traits that would predict or rule out applicants to become successful police officers (e.g., Detrick and Chibnall, 2006; Sanders, 2008; Lowmaster and Morey, 2012). Personality traits are, however, only a part of the picture. Research has shown that police officers’ behavior is determined by combinations of personality traits, such as extraversion and openness, and psychological states, such as frustration and depression (Varela et al., 2004).

As a last concern with current assessment practices, we point to the fact that testing is almost exclusively done during pre-employment (Marshall et al., 2017; 2020). Police organizations do not structurally monitor the psychological competence of police officers after they have been selected, and therefore fail to monitor changes due to training, stressful periods, traumatic events, and persistent mood states (Leigh Wills and Schuldberg, 2016). This would not be much of an issue if the traits assessed at pre-employment would remain stable over time. However, there is compelling evidence that personality traits may change during the course of life (see e.g., Roberts and Mroczek, 2008; Leigh Wills and Schuldberg, 2016). Due to sudden traumatic events or frequent exposure to stress, personality traits may change and develop over time. For instance, research has shown that resilience is a highly dynamic process that may vary according to time and circumstance (e.g., Rutter, 2012; Hu et al., 2015; Stainton et al., 2019). The concern with pre-employment assessment of psychological traits is thus that police organization measure properties once, while they are not stable over time. Current psychological testing methods fail to inform police organizations about the police officers’ risk of experiencing, developing, or abating psychological complaints after hire (Velazquez and Hernandez, 2019).

To summarize; psychological screening, aimed at assessing pure constructs (mostly personality traits) and limited to selection for employment, may have limited predictive value for psychological competence in practice of police officers during their lifelong career.

Baumeister et al. (2007) advocated direct observation of behavior to see how individuals actually behave, instead of introspective self-reports and questionnaire ratings. As Furr and Funder (2007) stated:

Any clue to a person’s psychology emerges from what that person does—what the person says, what the person writes, how the person responds to a questionnaire, how the person’s heart rate changes, which choices the person makes, what kind of intonations are present in his or her voice, or how the person reacts with others (pp. 277).

In line with this statement, we posit that direct behavioral observation is key in measuring the expression of competence in practice. Police organizations’ main interest should not be the psychological competence in itself, but rather the extent to which it does or does not facilitate the decisions and actions of the police officer. By placing police officers in situations that tax their psychological competence and measuring their decisions and actions with systematic behavioral observation, it is possible to assess the psychological competence they possess and can deploy in a practically relevant way.

Certain challenges accompany this type of assessment. The first challenge is that appropriate behavior in a certain situation can take on different forms. For example, while approaching a door with an unknown subject behind it, one police officer might approach the door cautiously, while another approaches it confidently. Both actions can be considered professionally adequate, and illustrative of competence. Oftentimes, there is not one single way of behaving that constitutes appropriate professional behavior and all other ways of behaving as inappropriate. Instead, a range of widely differing behaviors can potentially be indicators of competence. This provides a challenge when aiming to verify which behaviors are illustrative of psychological competence of police officers. So far, very little attention has been paid to methods to derive psychological competence from behavioral observation and therefore the intricacies to draw conclusions from the test results. Incorrect inferences can have far-reaching consequences for the police officers themselves, their environment and for society.

Second, taxing police officers’ psychological competence usually requires placing them in stressful situations (Andersen et al., 2016). It is highly unlikely that ideal, textbook behavior will be observed in stressful situations. Stressful situations induce various changes in behaviors, such as ineffective body movements and gaze behaviors (see for theoretical account supporting this suggestion: e.g., Oudejans, 2008; Nieuwenhuys and Oudejans, 2012, 2017; Renden et al., 2017). The challenge is to determine how much deviation from ideal, textbook, behavior can still be considered illustrative of competence, and how much deviation under stress is indicative of low psychological competence.

In this paper, we propose and showcase a methodology to develop an observational behavior assessment instrument that helps distinguish behaviors of police officers with high levels of psychological competence from those with lower levels of psychological competence. This type of testing can be applied to a wide range of moments when police organizations may want to measure psychological competencies of their police officers; for example, after stressful events, absence of leave due to psychological complaints, or as an annual psychological check-up. Within the scope of this study, we aim to provide a theoretical basis and practical guideline for police organizations for developing an observational behavior assessment instrument for psychological evaluation that could assist current demands in practice. As such, the study serves as inspiration for police organizations and other researchers to develop evaluation methods that move away from the currently flawed practices. It is beyond the scope of this study to prescribe one specific test including all empirical evidence and validation steps, which would be nonetheless an indispensable topic for further research. Because of the challenges outlined (i.e., a wide range of appropriate behavior and almost guaranteed deviations from ideal behavior in stressful testing circumstances) we pay particular attention to the interpretation of assessment. We have organized the paper in the following way: the methodology section will outline a step-by step proposed methodology to develop an observational behavior assessment instrument. In the section that follows, we illustrate the proposed methodology with a practical example. This example hopefully inspires police organizations and researchers to apply the methodology for their specific testing needs. In the last section, we discuss how the provided methodology contributes to practice and science.

## Methodology

We propose a methodology of building blocks to design an observational behavior assessment instrument. The building blocks are as follows: (1) the basis of the test, (2) scoring method, and (3) interpretation of scores (see Table 1). Within the building blocks, we propose specific steps to take, and suggest different methods or approaches for each step. Police organizations can use those steps that they consider useful in their practice and alter specific steps depending on the particular needs and purposes that they have with the assessment.

**TABLE 1:** An overview of the methodological steps to develop an observational behavior assessment instrument for psychological competence in police officers.

**The basis of the test**
Step 1: Establish the context of the observational behavior assessment instrument.
Step 2: Determine the psychological competencies of interest.
Step 3: Create relevant scenarios in which the psychological competencies are required.
**Scoring method**
Step 4: Elicit a diverse spectrum of behaviors.
Step 5: Select behaviors for the observational behavior assessment instrument.
Step 6: Design the scoring method.
Step 7: Select the assessors for observation.
**Interpretation of scores**
Step 8: Identify behavioral patterns in the test population
Step 9: Determine “desirable” and “problematic” behavioral patterns
Step 10: Match the police officers’ behavior with the “desirable” and “problematic” behavioral patterns

### The Basis of the Test

Making the basis of the test explicit provides police organizations with a clear view on the test’s objectives and what the test should look like in its final form. The basis is the cornerstone for methodological decisions about the scoring method, and interpretation of the scores (Kaslow et al., 2007).

#### Step 1: Establish the Context of the Observational Behavior Assessment Instrument

To establish the context of the test, three factors need to be discussed: (a) the situational context of the test, (b) the target group and the impact of the test, and (c) assessment level (Kaslow et al., 2007; Leigh et al., 2007). In Table 2, we suggest questions that police organizations could ask themselves to determine their context. The questions are based on the literature regarding direct behavioral observation (see Table 2 for references). The outcomes of the questions provide essential input for methodological decision-making in the further test development. For example, when the aim is to test behavior in stressful situations, the scenarios must contain characteristics that induce stress (such as uncertainty, possible threat) to elicit behaviors that occur in such situations (Renden et al., 2017). At the same time, the stress levels evoked in the scenario and the required behaviors must match the target group for which a test is intended. Ethical considerations can limit the extent to which stress is introduced, for instance when the target group consists of police officers in a recovery process after mental health complaints (Furr and Funder, 2007). Similarly, the behavior that the test aims to evoke should be aligned with the level of training of the test population. If police officers have been traumatized and not had training in shooting for a long time and yet the test requires shooting accuracy, then the level of execution of shooting is not indicative of psychological competence *per se*, but might merely represent training status. Thus, understanding and explicating the context of the test design will form the foundation for all following steps.

**TABLE 2:** Sample questions police organizations can use to determine their test context.

**The situational context of the test**
• Will the observations take place in a real-world setting or a laboratory setting? (Furr and Funder, 2007). • Does the test aim to capture behavior in a mundane situation, or are they intended to capture behavior in an unusual or extreme situation? (Furr and Funder, 2007). • Will the test be conducted in a specific situation, or should the test be applicable to a wide range of situations? (Suen and Ary, 1989).
**The target group and impact of the test**
• What implications does participation have for the participant? (Nock and Kurtz, 2005). • What implications do the results have for the participant? (Nock and Kurtz, 2005). • What is the experience of the participant? (Paoline et al., 2015).
**Assessment level**
• On what level of specificity will the behaviors be observed? (Furr and Funder, 2007; Furr et al., 2010). • What is the amount of interpretation to be required of assessors in evaluating behavior? (Furr and Funder, 2007; Furr et al., 2010). • What measures can be taken to reduce assessor error and assessor bias? (Hartmann et al., 2003).

#### Step 2: Determine the Psychological Competencies of Interest

To build a solid assessment, it is essential to determine the psychological competencies of interest (Fouad et al., 2009), and the scenario-contexts in which the psychological competencies are required (Furr and Funder, 2007). The objective of this step is therefore to determine the exact psychological competencies that police organizations aim to assess. Competencies of interest can be very specific, such as emotional resilience and information processing, for example if the assessment takes place after an absence due to psychological complaints (Ingram et al., 1998). In contrast, if police organizations carry out the test as a pre-employment selection tool, they may choose a wide range of psychological competencies of interest consisting of emotional, social, and cognitive factors, such as motivational and communications skills (e.g., Adang, 2011; Rajakaruna et al., 2017).

#### Step 3: Create Relevant Scenarios in Which the Psychological Competencies Are Required

The third step focuses on the interpretation of behavior in scenarios. Therefore, it aims to provide options for scenario development that police organizations can customize to fit their needs and competencies they are interested in, instead of suggesting fixed scenarios specific to scenario creation. Two key factors should be taken into account: (1) the scenarios must be suitable for demonstrating the competence, thereby distinguishing between people who show high levels of competence and people who (currently) show less competence, and (2) the scenarios are realistic and ecologically valid and therefore have predictive value for behavior in daily reality. In Table 3, we suggest questions, derived from the literature, that can support police organizations in the development of scenarios.

**TABLE 3:** Sample questions police organizations can ask when choosing and designing relevant scenario-contexts.

**The scenarios must be suitable for demonstrating the competence, thereby distinguishing between people who show high levels of competence and who (currently) show less competence.**
• Does the scenario provide a controlled and safe situation where police officers can make errors without danger to themselves, opponents, or bystanders? (Bertilsson et al., 2013). • Does the instruction before the scenario allow for different responses from police officers? (Koedijk et al., 2019). • Does the scenario consider experience of the police officers? (Paoline et al., 2015). • Does the scenario consider the norms and values in the immediate working environment of police officers? (Loftus, 2010).
**The scenarios are realistic and ecologically valid and therefore have predictive value for behavior in daily reality**
• Does the scenario provide a realistic environment that replicates what a police officer would expect to encounter in a real-life situation? (Andersen et al., 2016). • Does the scenario guarantee for experiencing levels of stress that enforce behavior that is representative for the situation? (Renden et al., 2017). • Does the scenario include representative attire for police officers and opponents? (Staller et al., 2017). • Does the scenario take into account ethical considerations that limit to which extent scenarios can be made demanding or stressful? (e.g., a setback in the recovery process for someone with mental health complaints) (Furr and Funder, 2007).

Perhaps one of the main challenges in scenario development is to make the scenario specific enough for the competencies of interest, and yet realistic enough to be ecologically valid. To maximize the chance that competencies are displayed without compromising the realism and open structure of the scenarios, practical experience and expertise must be an integral part of scenario development. We suggest two main strategies to do so. The first strategy is to seek out retrospective information from officers about situations they have encountered and responded to (e.g., Lavoie et al., 2016; Koedijk et al., 2019). Methods to collect these situations include for example consulting case reports, organizing focus groups, and interviewing experienced officers. Police organizations can borrow from published literature. For instance, Bertilsson et al. (2019) developed scenarios in which the participants performed six different tasks, including no threat, moderate threat, and high threat scenarios. Similarly, Giessing et al. (2019) presented extensive examples of high stress and low stress scenarios and showed that high-realism scenarios afford an opportunity to investigate and experience stress responses.

A second strategy is the observation of behaviors in the working environment itself. A task analysis can be conducted to collect the occurrence of situations on duty and the response in these situations by police officers (see for examples of task analysis: e.g., Anderson et al., 2001, 2005). Both strategies render realistic situations and responses, which should then be explicitly tailored to align with the psychological competencies of interest. This means that the situations should tax the psychological competencies of interest, and effective, realistic, responses should be contingent upon levels of the psychological competencies (Andersen et al., 2016).

### Scoring Method

#### Step 4: Elicit a Diverse Spectrum of Behaviors

The fourth step is to compile an extensive list of behaviors that may occur in the scenarios and that may be indicative of the psychological competencies of interest. The question to be answered in this step is “what to observe?.” This (usually) involves behavioral sampling (Suen and Ary, 1989). Police officers may display countless behaviors in the scenarios, ranging from observable gross motor movements to behaviors not easily detectable (such as avoiding eye contact). One can collect and select relevant behaviors by various methods. Some examples are Delphi Methods and expert panels (see for example Nevo and Chan, 2007; Jeste et al., 2010), focus groups and individual interviews (see for example Clifton and Handy, 2003; Ryan et al., 2014), or ethnography (see for example Passos et al., 2012). The challenge is to collect ecologically valid behavior. As pointed out previously within scenario development (see Step 3), practical experience and expertise are indispensable to do this. Police organizations can also borrow from recent examples of best practices to judge their officer’s performance based on an observable and detailed set of essential policing behavior. Bertilsson et al. (2019) defined a systematic observation protocol with questions of how police officers handle the situation in terms of perception, communication, voice control, motor control, spatial and temporal tactical implementation. In a similar vein, Huhta et al. (forth coming) identified six behavioral dimensions to judge police performance in critical incidents simulations, such as operational flexibility, initiative, and target-oriented behavior. Both studies showed the successfulness of using predesigned behavioral performance aspects, relevant in police situations, to collect a detailed set of information about when and how a police officer was affected by stress.

#### Step 5: Select Behaviors for the Observational Behavior Assessment Instrument

The fifth step is to select, from the extensive list of behaviors, the behaviors to include in the assessment instrument. The list of behaviors established in Step 4 helps determining what to observe, but such a list can be too substantial to use. To create a useable instrument, a further selection of behaviors may be required (Hintze et al., 2002). The literature offers multiple guidelines to support the appropriate selection of behaviors in assessment development:

• Decisions should be made on the specificity of the behaviors to include in the instrument (Bakeman, 2000; Furr and Funder, 2007). Behaviors to include in the instrument can be general, such as “acts socially uncomfortable,” and “avoidance behavior,” or specific, such as “appoints someone,” “hands are shaking,” and “grabs handcuffs.”
• Decisions should be made on the amount of psychological inference required by the assessors (Nock and Kurtz, 2005). The level of specificity on which the behaviors are displayed is linked closely to the amount of psychological inference required by the assessors (Bakeman, 2000; Furr and Funder, 2007). Specific behaviors such as grabbing handcuffs require minimal inferences regarding the meaning of participants’ behavior. In contrast, behaviors on a macro level, such as acting socially uncomfortable, require much interpretation by the assessors that can diverge widely among assessors. Therefore, the identification, definition and evaluation of discrete, observable behavior should be as specific as possible to avoid discrepancies in the interpretation of behavior (i.e., inter-rater disagreement).
• Behavior definitions should be checked whether they are unambiguous such that an assessor could read it and define it accurately (Hintze et al., 2002).
• Ideally, pilot studies with the assessment instrument should be run to select behaviors on the basis of analysis of the collected data, that is a data-driven selection of behaviors from the long-list. Particular behaviors to omit are behaviors that are not observed at all, observed in all cases, and behaviors that are always observed together. If all participants displayed a particular behavior, or none of them displayed the behavior, this behavior is considered ineffective for determining behavioral patterns in a population (Suen and Ary, 1989).

#### Step 6: Design the Scoring Method

Police organizations traditionally use rating scales that inform police officers through single construct scores [see, e.g., MMPI scales (Inwald and Shusman, 1984; Shusman et al., 1984)]. Police organizations should dismiss these practices and choose a scoring method that provides a more detailed and rich account of individual officers’ psychological competence. By adopting the concept of behavioral observation as a scorings method, we hope to set a good example of a detailed and rich evaluation that informs about psychological competence as required in practice.

Two choices have to be made with regards to the behavioral observation scoring method: (a) how observation and scoring will take place, and (b) how the behaviors are presented in the scoring instrument for easy scoring. As for the method of scoring, police organizations should choose which information is most relevant for assessment interpretation. In the observation procedure, assessors mark the occurrence of behaviors from a list of potential behaviors. But which aspect of behavior is most indicative of the psychological competencies of interest? Would that be presence or absence of behavior (e.g., BASC, Reynolds and Kamphaus, 1998), frequency of behavior (e.g., O’Sullivan et al., 2002), intensity or duration of behavior scale (see for applications of scoring methods to do so: e.g., Latham and Wexley, 1977), etcetera. The choice of method of scoring is related to the inference required from assessors (see also Step 5). For example, with a Likert scale, the assessors will have to judge whether they think a behavior has been showed by the participant often or “to a high degree.” When the behaviors have clear start and endpoints, it should be considered to use occurrence or frequency scoring. When the behaviors are continuous and take longer, police organizations may consider scoring the duration of a behavior during the scenario or specific time interval (Hintze et al., 2002).

One way of organizing the behaviors in the scoring instrument for ease of scoring would be to cluster behaviors around the timeline in which they are expected to occur (e.g., Volpe et al., 2005; Furr et al., 2010). Other alternatives to categorize behaviors in the scoring instrument are type of behavior (e.g., communications, and non-verbal expression), the frequency of occurrence, and to what extent the behaviors are typical for the scenario (e.g., most distinctive behaviors first).

#### Step 7: Select the Assessors for Observation

The seventh step is to determine who qualifies as an assessor. The importance of a careful selection of assessors for accuracy and agreement has been well documented (Lievens, 2001). To select assessors, three types of considerations should be taken into account: (1) conceptual considerations, (2) performance considerations, and (3) practical considerations (see e.g., Hartmann et al., 2003; Nock and Kurtz, 2005).

##### Conceptual considerations

The extent to which assessors are sensitive to bias should be addressed. The main bias in assessment is usually associated with assessors’ expectancies, prejudices, and information-processing limitations (Berendonk et al., 2013). Cone (1998) described two crucial factors for controlling assessor bias. First, the amount of psychological inference required by the assessors should be considered (see also Step 5). A behavioral coding that requires minimal inferences regarding the meaning of participants’ behavior is less affected by assessors’ view how participants should behave than when a lot of inference is needed (Cone, 1998). In the latter case, it is desirable to assign assessors who are trained in suppressing bias and experienced in making inferences. Additionally, and perhaps obviously, assessors must be familiar with the construct being tested and accompanying behavior. With increasing familiarity in the actions required to appropriately fulfil the demands of the scenario, assessors may become more efficient in processing of observational information (Berendonk et al., 2013).

##### Performance considerations

The complexity of scoring procedures can vary widely. This variation makes it important to establish the need for training of assessors in the basics of the scoring procedure (Furr and Funder, 2007). The training of assessors can include issues as how to observe behaviors, how to recognize behaviors, and how to interpret behavior. In addition, Cone (1998) identified that motivation seems essential in assessors’ performance. Police organizations should arrange the observational setting in such way that assessors feel familiar with the material and the workload.

##### Practical considerations

The extent to which assessors are sufficiently available and associated costs (Cone, 1998). As an example, a police organization may have to choose to use external assessors from outside their organization (external assessors) or from within (internal assessors). The choice for an internal assessor has the advantages that their presence may be less obtrusive, and their access to infrequent behavior. On the other hand, they may be less dependable and more subject to bias than external assessors may be (Cone, 1998).

### Interpretation of Scores

In the steps proposed thus far, we addressed the psychological construct, the test-context, the relevant behaviors, the observational assessment instrument, and the instructions for the assessors. The last building block in our proposed methodology is the interpretation of the scores. The matter of interpretation of scores has received little attention in the literature, and is as such underexposed. The aim of this block is to provide a method of how police organizations can derive psychological competence from the observation of behavior in a useful and interpretable way. Due to the limited literature on the topic, the recommendations we provide for these steps are strongly guided by our practical experience in assessment and scoring design, and closely intertwined with the example provided in the next section.

#### Step 8: Identify Behavioral Patterns in the Test Population

A fundamental question towards interpretation of observational data is whether different groups in the test population (that is groups with different levels of psychological competence) show distinctively different behaviors during the experimental scenarios. The knowledge, skills, and attitudes of police offers materialize as a set of actions (Cherniss and Boyatzis, 2013). When these actions are a pattern of behavior that generalizes across settings and stimuli, they form competencies (Boyatzis, 1982). This suggests that the identification of behavioral patterns is the way forward to draw conclusions about psychological competence.

A latent class analysis (LCA) is considered the most common approach to identifying behavioral patterns (e.g., Laska et al., 2009; Tabacchi et al., 2018). Goodman (1974, 2002) established the LCA theoretical framework and demonstrated its effectiveness in identifying latent classes (or unobserved groups) within the population. These latent classes are defined as groups within a population that show a distinctive, mutually exclusive pattern of answers to categorical variables. With a view to development of observational behavior assessment instrument, instead of patterns of answers to categorical variables, we use patterns of observed behaviors. For instance, within a population of police officers, officers who display certain behaviors, such as frequently asking questions to the instructor, keeping a safe distance from the suspect, drawing a weapon, and approaching the suspect cautiously, would belong to a distinctively different latent class than officers who do not display any of these behaviors, or officers who draw a weapon and approach the suspect cautiously, but do not ask questions frequently and do not keep a safe distance. For a further illustration of LCA to identify behavior patterns, we refer to the practical example in the next section.

#### Step 9: Determine “Desirable” and “Problematic” Behavioral Patterns

The next step is to determine desired and undesired (or problematic) behavioral patterns. The aim is to establish which behavioral patterns are related to police officers’ high levels of psychological competence levels and which patterns point to problems with psychological competence. Behavioral patterns that are neutral in terms of police officers’ psychological competence levels may also arise from the analysis. It is our aim to provide an example of the methodology, not present the pilot study in full; therefore further details on the statistics are beyond the scope of this example.

It will depend on the aim of the test (see section: basis of the test) whether police organizations are mainly interested in the presence of desirable patterns (e.g., to establish level of training, or candidates for promotions), or in the absence of problematic behavioral patterns (e.g., to determine whether a police officer sufficiently recovered from trauma to rejoin the workforce). To infer whether behavioral patterns are desirable or problematic, several procedures can be applied that are typical for determining validity of tests (for a contemporary view on validity testing and a summary of seminal work on validity see Lissitz and Samuelsen, 2007). These procedures relate the established behavioral patterns with measures of other characteristics of police officers that relate to psychological competence (e.g., job performance, mental health status, stress reactivity, etc.). In the practical example in the next section, we share how we used other measures of psychological competence for the purpose of identifying problematic behavioral patterns.

#### Step 10: Match the Police Officers’ Behavior With the “Desirable” and “Problematic” Behavioral Patterns

The tenth step is to arrive at a conclusive method to match the behaviors that are observed in a police officer during the observational behavior assessment with the desirable or problematic behavioral patterns identified through Steps 8 and 9. The result of the test should represent the extent to which the behavior of a police officer in the assessment matches with behavioral patterns that are identified as desirable or problematic. The criteria below must be taken into account in determining the strength of association between the observed behavior and the established behavioral patterns. It may be helpful to keep in mind that if we refer to behavioral patterns we mean the patterns that are established with a large test population, and can be considered as a scoring mold or reference norm. If we refer to observed behavior, we mean the behavior of an individual police officer during the test, as scored by assessors.

The strength of the association between an observed behavior and a behavioral pattern stems from two things, likelihood and exclusivity. With the likelihood, we mean the chance that a behavior occurs within a behavioral pattern. If a police officer demonstrates a behavior that is almost guaranteed to occur within a behavioral pattern, than this heightens the association between the observed behavior and the behavioral pattern strongly. If a police officer demonstrates a behavior that possibly, but not always, occurs within the behavioral pattern, then the association between the observed behavior and the behavioral pattern should be considered as less strong. With exclusivity, we mean the extent to which a behavior occurs exclusively in one behavioral pattern but not in other behavioral patterns. If a police officer demonstrates a behavior that is almost exclusively to occur in one established behavioral pattern this suggests a strong match between the observed behavior and the behavioral pattern. If the behavior can occur in more than one behavioral pattern, in other words is not so specific or characteristic for the behavioral patterns, then the association between the observed behavior and the behavioral patterns is weaker.

By evaluating the strength of association of the observed behavior with desirable or problematic patterns, a conclusion can be drawn about the test result (see the practical example in the next section for a detailed example of an evaluation of the test result).

## Practical Example

The methodology that we present in this paper was applied in the development of an observational behavior assessment instrument for the Dutch National Police. We will particularly elaborate on the interpretation of the scores, thus how police organizations can derive psychological competence from the behavioral observation. Therefore, we present Step 1 to 3 and Step 4 to 7 (basis of the test, scoring method) together and briefly. We will then present step 8 to 10 (interpretation of scores) in greater detail.

### The Basis of the Test

To illustrate the basis of the assessment in our project, we briefly describe the situational context of the assessment, the target group, and assessment level. The assessment aims to identify police officers’ readiness to retrieve their service weapon after they handed it in due to temporary psychological issues such as suffering from burnout syndrome or traumatic experiences. The assessment intends to capture behavior in different work-related scenarios that differed in level of threat, and that are indicative of readiness to retrieve the weapon.

We formulated two requirements concerning the assessment level (in line with our proposed methodology): (1) the test result should be based on behavioral patterns displayed by police officers in the scenarios, and (2) we need a test result in which the behavioral patterns of police officers can be matched to high and low psychological competence. Collectively, the assessment should indicate whether there are any objections for retrieving the service weapon, as such we were interested in absence of problematic behavioral patterns.

The Dutch National Police selected three psychological competencies of interest: (1) Emotional resilience defined as the ability to be emotionally balanced, to have self-confidence and emotional self-insight to recognize limits, and regulate emotions, (2) Pressure resistance is defined as adequate performance in hectic, stressful and threatening situations, the control of one’s impulses, and the capability to operate under pressure to judge reality adequately, (3) Information processing defined as the ability to distinguish between relevant and less relevant information and interpret information correctly.

We, led by experts of the police organization, constructed five scenarios that differed in the levels of threat and the number of unexpected events. For example, a highly stressful scenario could follow a relatively calm scenario, and a controlled and procedural scenario could follow a scenario where officers had to respond as quickly as possible to an unexpected event.

In the remainder of this manuscript, we will elaborate on one of the five scenarios only. In this vein, we can illustrate the further steps of the methodology by means of a practical example, without disclosing the full test protocol, which would be neither desirable nor functional. The police officer receives the following instructions from a police instructor: “There is a person behind the door with a handgun. Approach the door and act according to what you think the situation asks for.” When the police officer asks for more information, the instructor repeats the same instruction. The police officer then is expected to approach the door. When the officer knocks on the door and asks the person behind it to make themselves known, there is no response. When the police officer opens the door, there is a person sitting calmly in a chair with a handgun visible in his belt. His hands are visible and he fully cooperates with any instruction the police officer gives. The scenario is stopped when the police officers starts to arrest the person (i.e., handcuffing). The instructor stops the scenario and starts a short, systematic evaluation by asking the police officer which instructions they received before approaching the door and which actions they performed during the scenario.

### Scoring Method

We created the scoring method on the basis of focus groups. Police instructors, operational police officers, and police officers in reintegration, for example officers recovering from burnout syndrome, participated in the focus groups. The aim of the focus groups was twofold: First, to compile a list of behaviors that are likely to occur during on-duty work and particularly in the assessment scenarios (the long list of behaviors); Second, to establish behaviors that police officers believed to be indicators for high and low levels of emotional resilience, information processing, and pressure resistance (a first aid for the selection of behaviors to be included).

**FIGURE 1:**
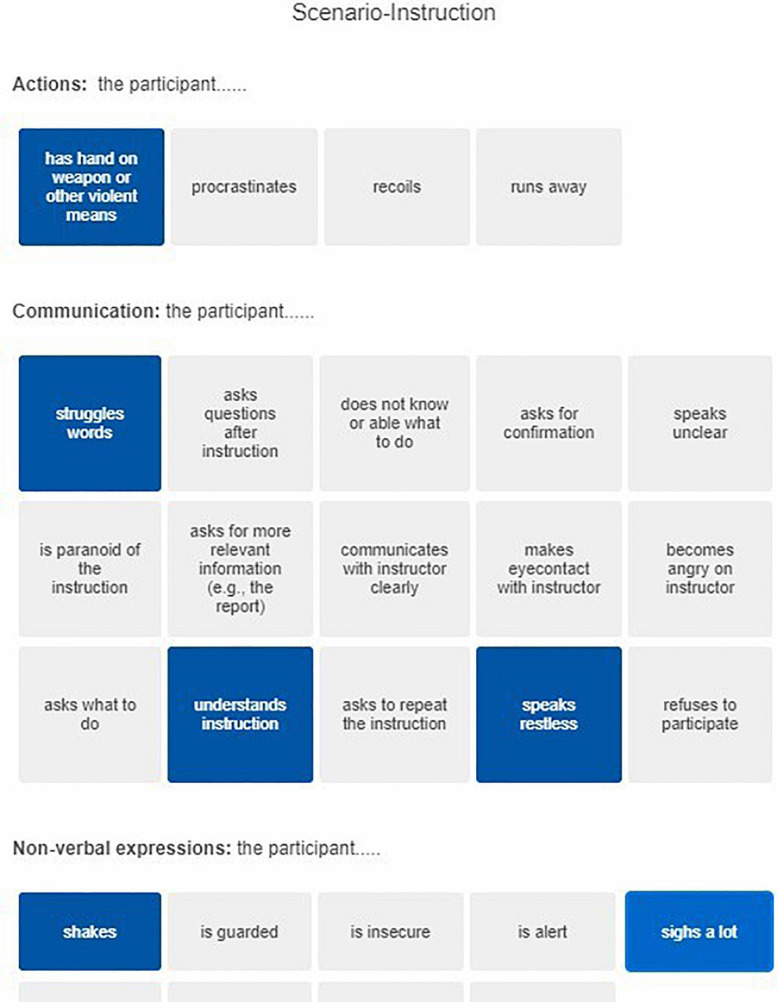
Example page of the assessment instrument used during the behavioral assessment of the experiment. The picture shows how assessors could select/tap behaviors during the observation.

The focus groups rendered an extensive list of behaviors, which we organized by phase in the scenario (four phases: instruction, approach to the door, after the door, and evaluation) and type (actions, communication and non-verbal expressions) and programmed in the survey software Qualtrics to create a scoring instrument. This organization of behaviors allowed assessors to easily select observed behaviors specific to each phase and enabled the researchers to evaluate changes in behavior across and specific to phases. Figure 1 shows an example page of the scoring instrument used during the behavioral assessment.

The assessment instrument was used during a pilot study to further develop the scoring method. Participants were police officers who were allowed to carry their service weapons, and not yet the target group of police officers who had handed in their service weapons. A sample of 115 healthy operational police officers of the Dutch National Police took part in the pilot study. The pilot took place in a training facility of the Dutch National Police. Police officers were equipped with training gear consisting of a blue gun (training gun similar to a service weapon), non-lethal training ammunition, which creates no impact or sound but makes it possible to determine whether a shot was fired, pepper spray, and handcuffs. Police officers performed the five scenarios of the protocol consecutively.

Two assessors (one police instructor and one psychologist) indicated the occurrence of behaviors (i.e., selected/tapped the observed behavior from the list of behaviors) during the scenarios (see Figure 1). After each scenario, the assessors independently gave a score for each psychological competence (Emotional resilience, Pressure resistance, and Information processing). We chose police instructors as assessors because of their familiarity with the actions required to appropriately fulfill the scenarios’ demands, and their experience with both formative and summative assessment of competence. We chose psychologists as additional assessors because of their familiarity with psychological competencies and their expertise in unbiased observation of behavior. Within four weeks after participation in the pilot, the police officers filled out the mental health short form (Keyes et al., 2008), and self-ratings for their psychological competencies. They were asked to rate their subjective level of each psychological competency during the last month, and at the specific day they participated in the pilot. We do not consider psychological competence to be varying on a one day to the next basis, but we hoped that this way of asking would enhance honest scoring. It allowed police officers to present themselves as competent in general, but “admitting” that at the day of testing they were not at their best.

All in all the pilot resulted in scores of behaviors that were observed by the two assessors in the scenarios, ratings of the psychological competencies displayed in the scenarios according to the assessors, self-rating of psychological competencies of participants, and mental health status of participants as assessed by the mental health short form.

### Interpretation of Scores

#### Step 8: Identify Behavioral Patterns in the Test Population

To be able to interpret the observed behaviors in a way that is meaningful to assess the psychological competencies of interest (Emotional resilience, Pressure resistance, and Information processing) we wanted to identify behaviors or behavioral patterns that may relate to the competencies. We performed a latent class analysis (LCA) on the behavioral data to identify whether latent classes seemed present in the test population that show distinctively different behavioral patterns during the scenario. The technical details of LCA are beyond the scope of this practical example. Instead we will illustrate the way we worked with LCA using behaviors that were observed in the “after the door” phase of the scenario. LCA distinguishes latent classes (groups within the test population that are characterized by specific behavioral patterns) and calculates probabilities that a certain behavior occurs by people belonging to each latent class. For a more guidance on the description and functions that LCA may serve in police officers’ behavioral assessment and resources to learn how to conduct a LCA, we refer to the Supplementary Material.

**FIGURE 2:**
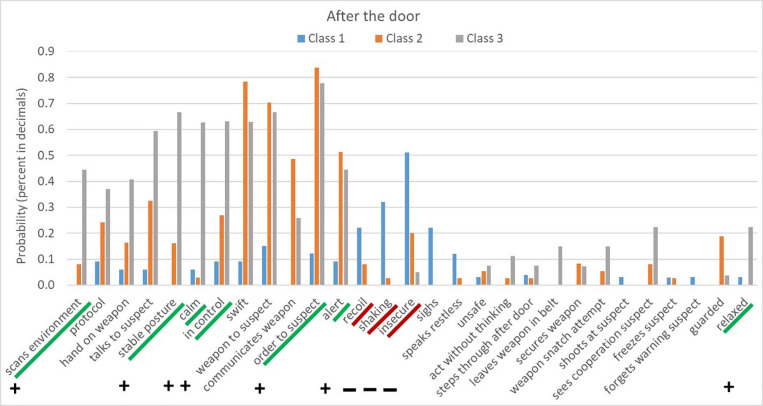
The expected probabilities of the behaviors occurring in each of the three latent classes during the “after the door” phase. The figure also shows which behaviors are positively (underlined in green with plus sign) and negatively (underlined in red with minus sign) associated with psychological competence (see step 9). For behaviors that are not underlined no significant difference was found in psychological competence between the group that displayed the behavior and the group that did not.

Figure 2 shows an example of the probabilities of the behaviors for three different classes. For instance, the behaviors ‘scans environment,’ ‘follows protocol,’ ‘hand on weapon,’ ‘talks to suspect,’ ‘stable posture,’ ‘in control’ are least likely to be displayed by latent Class 1 and have a much higher likelihood to be displayed by latent Class 2 and 3 (see Figure 2). To further help interpretation of Figure 2: if we assume that a certain participant displays behavior that correspond with the behavioral pattern in Class 1, the participant has a probability of.13 (out of 1) to give orders to the suspect behind the door. If we assume that the participant displays behavior that correspond with the behavioral pattern in Class 2, this probability is.85 (and.75 if the participant displays behavior that correspond with the behavioral pattern in latent Class 3).

We contain that behaviors that show a minimal difference in probability across latent classes (i.e., less than 5% probability difference, for example) do not meaningfully distinguish behavioral patterns from each other. Behaviors such as ‘unsafe conduct’, ‘secures weapon’ (Figure 2) had a low likelihood to be displayed by any of the latent classes. Therefore, these behaviors do not provide insights into the behavioral difference of the three latent classes. Similarly, if probabilities of a behavior are high for all three classes, the specific behavior is not distinguishing between classes, and is thus irrelevant for our purposes.

#### Step 9: Determine “Desirable” and “Problematic” Behavioral Patterns

The LCA rendered latent classes for each phase of each scenario. The key question was whether these classes, with corresponding patterns of behaviors, were related to psychological competencies. In other words, does the latent aspect that clustered the behavior as a pattern actually have to do something with psychological competencies.

For each behavior, we conducted independent-samples *t*-tests to compare the scores of psychological competence (the assessor scores, the participants self-ratings and the mental health scores) between participants that had displayed the behavior during the pilot, and participants that had not displayed the behavior. The results indicated that, indeed, the latent classes seemed to relate to psychological competencies. We found that participants who demonstrated behaviors that were characteristic for specific latent classes (i.e., had a high probability to occur for a specific latent class, and not for others) significantly differed in psychological competence scores from participants who did not demonstrate these behaviors. It is our aim to provide an example of the methodology, not present the pilot study in full; therefore further details on the statistics are beyond the scope of this example.

To further explore and interpret the patterns, we presented the results of the latent class analysis and independent *t*-tests to experts (police instructor and psychologist). Figure 2 shows an example of the representation of results, and incorporates the results of the *t-*tests. For behaviors that are underlined in green, we found that participants who demonstrated the behavior had higher psychological competence scores than participants who did not demonstrate the behavior. In contrast, the behaviors underlined in red indicate that participants who demonstrated the behavior had lower psychological competence scores than participants who did not demonstrate the behavior. We discussed the results for all phases (instruction, before the door, after the door, etc.) of all scenarios, and established which latent class(es) constituted of “problematic” behavior patterns, that are associated with lowered psychological competence. In Figure 2, we identified the behavioral pattern in class 1 as “problematic” In short: if a police officer demonstrates the undesirable behavior pattern in the assessment, this might raise objections or concerns for retrieving the service weapon.

#### Step 10: Match the Police Officers’ Behavior With the “Desirable” and “Problematic” Behavioral Patterns

As the last step, we wanted to determine the match between a subjects’ behavior during the assessment and the identified behavioral patterns of the latent classes. To do so, we refer back to Figure 2 with the classes and probabilities. We will compare the behavior that an individual candidate has shown with the Figure’s information. As explained in Step 10 of the methodology section, the matching criterion is the strength of the association (i.e., likelihood and exclusivity) between observed behavior and the behavioral patterns. To illustrate these concepts, we suppose that the police officer pointed the weapon at the suspect during the experimental scenario, was alert, had a stable posture, and was in control. The police officer showed behaviors that have a high likelihood to occur in class 3 (see Figure 2). The behaviors of pointing the weapon towards the suspect and being alert are likely to occur in more than one behavioral pattern, namely also in class 2. This means that these behaviors are not so specific or characteristic to a behavioral pattern (i.e., low exclusivity), and are therefore less meaningful in determining the match of police officer’ behavior and a behavioral pattern. The other behaviors, namely stable posture and in control, are only likely to occur in class 3 and not in class 1 and 2 and are, therefore, highly exclusive for class 3. As such, these behaviors have both a high likelihood and exclusivity and thus suggest a strong match between the observed behavior with the ‘desirable’ class 3. The association between the officers’ behavior and the behavioral pattern class 3 is strong, suggesting that the officer belongs to class 3, which is not a problematic class.

The assessment aims to eventually determine whether there should be doubts about the psychological competencies of the assessed officer, thus raising objections to retrieve the service weapon. Therefore, matches between the behavior of the subject and undesirable behavioral patterns are of particular interest. To take another example, we now suppose that during the experimental scenario, the police officer was shooting at the suspect, sighing and acting insecure. The police officer showed behavior that is only likely to occur in class 1. The behaviors of shooting the suspect and sighing have a low likelihood that these behaviors will occur (see Figure 2). This makes the association with class 1 less strong, and these behaviors less meaningful to determine the match. Insecure behavior, however, has a reasonably high likelihood to occur in class 1. As such, this behavior has both a high likelihood and exclusivity and thus suggest a strong association between the observed behavior of the police officer and the ‘problematic’ class 1. For more guidance and examples of how police organizations can determine the association between police officers’ behavior and the classes, we refer to the Supplementary Material.

The assessment and particularly the way of displaying scores will, in the last phase of assessment development, be further developed and tested with the actual target population; police officers who wish to retrieve their service weapon after they handed the weapon in due to psychological concerns (Work in progress, not described here). The accuracy and usability of the scoring instrument with a visual display of the strength of association of observed behaviors with behavioral patterns determined by LCA will be of particular interest in that evaluation.

## Discussion

Current practices for assessment of psychological competence of police officers have limitations, namely that they mostly focus on personality traits instead of competent behavior and that they are almost solely used for selection-for-hire purposes. This paper moves away from these current flawed practices and aimed to promote the alternative of observational behavior assessments to test a more detailed, rich account of an individual officer’s psychological competence, and for multiple purposes (e.g., selection, after traumatic events, absence of leave due to psychological complaints, or as an annual psychological check-up). To do so, we proposed a methodology to develop an observational behavior assessment instrument and illustrated the methodology with a practical example.

The ideal assessment would use both direct observations of behavior and measurement of psychological processes that mediate and produce those behaviors (Baumeister et al., 2007). Nevertheless, researchers seem to have turned away from behavior observation. Baumeister et al. (2007) attribute this to different (publication) standards that seem to apply to behavioral assessment and psychological states or traits assessment. They aptly stated:

“Behavior by itself is regarded as only a beginning, an unsolved puzzle. Meanwhile, however, a study of inner process without behavior is acceptable. When confronted with a study reporting behavior but not inner process, reviewers will immediately ask, why did this happen? Researchers need to show what goes on inside. But when confronted with a study reporting inner process but no overt behavior, reviewers almost never ask, “would this actually alter behavior?”(pp. 401).

This paper aims for the middle ground in the dilemma above by proposing an observational behavior assessment instrument that is indicative of psychological competencies in police officers, thus aiming to make judgements on inner processes (the psychological competencies) by assessing how they are expressed in action (behavioral assessment). We posit that direct behavioral observation can be key in measuring the expression of competence in practice, and that competence in practice is what police organizations should care about. The field would benefit from developing more methods that focus on and gather information about a wide range of behaviors reflecting psychological competence.

Behavioral assessment is not new in psychology; multiple studies have provided us with a basis to depart from (e.g., Bakeman, 2000; Thompson et al., 2000; Nock and Kurtz, 2005; Furr and Funder, 2007; Furr et al., 2010). These studies discuss important aspects such as the observational context to use, which behaviors to assess, and how to develop the assessment design. The actual implementation and evaluation of behavioral assessment has received considerably less attention. Furr and Funder (2007) did provide insight into the implementation and evaluation of direct behavioral observations. They offer an extensive and insightful example of how to use various statistical techniques to make judgments about psychological evaluation. Similarly, this study also provides an example of the interpretation of behavior. In contrast, we do not describe a detailed statistical procedure, but outline a step-by-step methodology for police organizations to measure and evaluate behavior in a meaningful way to assess psychological competencies.

Furr et al. (2010) stated that methodological decisions in developing an observational behavior assessment instrument should rest on a blend of conceptual and practical considerations. We share this line of thinking and were concerned with assessment development that is useful in practice. An essential requirement here is functionality, something that works. An epistemology that sits well with this is pragmatism, in which the researcher considers the methodological approach that works best for the particular research questions being investigated (Tashakkori and Teddlie, 1998). Hutter et al. (2016) described the pragmatism paradigm with the question “what works for which outcomes, and how?” (p. 64).

Our pragmatism speaks from the choice to present a methodology with various options, rather than a strict or specific protocol. It depends on the function that police organizations have for the assessment how the assessment should be fleshed out and which of the provided options are chosen. In other words, police organizations can choose aspects or methods that they find most relevant, and be guided by what works best for their testing practice. In this pragmatic line of thinking, the practical example should not be seen as a ready-made replicable protocol for assessment, but as an example of how the development for a specific desire of the Dutch National police practically worked out. For instance, in our practical example, the methodology is applied in assessment to determine whether someone is back on a sufficient level after this has not been the case for a period *(retrieving the service weapon).* Safety organizations may also consider using the methodology as inspiration to develop a screening assessment to determine whether someone is psychologically competent to enter in the program, or to determine whether psychological competence levels are maintained after the initial training.

There is ample room for further research in observational behavior assessments within police organizations. While this study presents a good starting point to decide how much deviation from ideal, textbook behavior can still be considered illustrative of competence, and how much deviation under stress indicates low psychological competence, more information should be obtained to improve the knowledge base of psychological competence in police officers. Because this study investigated behaviors associated with high levels of psychological competence and behaviors indicative of lower levels of psychological competence in a population of healthy operational police officers, future research should determine whether behavioral display might differ in police officers that show regular stress-responses and officers who suffer from psychological problems. For instance, incorporating a participant pool with police officers in reintegration might provide insights into this behavioral differentiation. This comparison might provide insight into excessive behavioral responses to stress and can, in the future, provide pointers for assessors to identify the distinction between normal stress reactions and excessive stress.

The insights presented in this study may be of assistance for police organizations to change their way of testing psychological competence. Follow-up steps should be taken when organizations intend to develop a specific and ready to use test. It will be required to build further on the theoretical basis and practical guidelines provided in this study. For instance, maintaining a database with scores from the target group to validate the scoring method, research into the reliability of the assessment, and finally research into the ease of use of the instrument and test experience of participants themselves.

## Conclusion

This manuscript intended to contribute to the knowledge and methods base for police practice concerning assessment of psychological competence, particularly through behavioral assessment. As such, we hope to provide an impetus to new ways of assessing that fit well with practice. We hope this manuscript provides police organizations with a methodology to perform scientifically informed observational behavior assessment of their police officers’ psychological competence and inspire additional research efforts in this important area.

## Data Availability Statement

The datasets presented in this article are not readily available because the data is confidential, as required by our ethics protocol. Requests to access the datasets should be directed to MK, m.koedijk@vu.nl.

## Ethics Statement

The studies involving human participants were reviewed and approved by Vaste Commissie Wetenschap en Ethiek, Vrije Universiteit Amsterdam (VCWE-2019-097). The patients/participants provided their written informed consent to participate in this study.

## Author Contributions

MK, PR, RH, and LK led the data collection and performed qualitative and quantitive analysis. MK wrote the first draft of the manuscript. PR, RH, and RO supervised the research process and contributed to the manuscript revision. RH wrote sections of the manuscript. All authors contributed to the conception and design of the study and read and approved the submitted version.

## Conflict of Interest

The authors declare that the research was conducted in the absence of any commercial or financial relationships that could be construed as a potential conflict of interest.
