# Supplementary material for: Observational Behavior Assessment for Psychological Competencies in Police Officers: A Proposed Methodology for Instrument Development
Source: Front Psychol. 2021 Feb 26;12:589258. doi: 10.3389/fpsyg.2021.589258 (PMC7959728; doi:10.3389/fpsyg.2021.589258)
Supplement: Supplementary file 2 [file Data_Sheet_1.pdf]

frontiers

Supplementary Material

1 General description of Latent Class Analysis (LCA)

To illustrate the functions that LCA may serve in behavioral assessment of police officers, we will describe LCA using an example. Suppose that a police organization aimed to identify police candidates with good communications-skills as part of their pre-employment selection. They determined seven possible indicators for communication: making eye contact, asking questions, attentive body posture, listens carefully, rushes the conversation, relaxed, open stance and speaks restlessly.

The assessors of the police organization notice during the pre-employment selection days that certain patterns of behavior seem to exist. They notice that certain candidates make eye contact and ask a lot of questions, whereas others listen carefully and have an attentive body posture. Both types of candidates may or may not rush the conversation and may or may not have a relaxed stance. The selection committee would like to know whether the patterns that assessors seem to notice exist, whether perhaps other patterns are present but have gone unnoticed thus far and whether any patterns of behavior can inform them on candidates that will be strong communicators or weak communicators.

The selection committee decides to conduct a behavioral observation to systematically collect data on behaviors displayed by candidates during a role-play with a suspect. The simplest way to investigate if the candidates who ask questions also make eye contact is to tally every time the behaviors co-occur. Table 1 depicts the results of the behavioral observation and shows that two police candidates both asked questions and made eye contact, one candidate made eye contact but did not ask a question, and three candidates did not make eye contact, nor did they ask questions. Indeed these results may point to a pattern of eye contact and asking questions co-occurring. After the observation, the selection committee discovered more combinations of behaviors that co-occurred frequently. For instance, four of the six police candidates listened carefully and rushed the conversation. Clearly, with such a small group of candidates, these 'patterns' are not very solid and may be the result of coincidence rather than an actual underlying pattern. To establish more reliable patterns much more participants would be needed. Additionally, in many cases a lot more behaviors than just seven will be of interest. Moreover, there may be more than two patterns present in the behaviors of candidates. Trying to establish patterns with more participants, more behavioral indicators, and potentially more patterns present, is soon becoming much too complex for this simple 'tallying co-occurrence of behaviors' method and interpreting the frequencies of co-occurrence.

Conducting a Latent Class Analysis (LCA) can then offer a solution. In a similar vein as the manual tallying of combinations of behavior but automated, the LCA identifies patterns based on observed behavioral indicators and their co-occurrence. Technically speaking, LCA uses parameters of logistic regression to estimate the likelihood of binary outcomes to occur when a specific condition is fulfilled. In this way, a LCA distinguishes "latent" classes within a test population characterized by a

specific behavioral pattern. "Latent" means unobserved. The behavioral indicators that are observed in the behavioral observation may be related due to unobserved, underlying influences. For example, the behaviors asking questions and making eye contact may co-occur because extraverted candidates display both behaviors, and therefore they co-occur. Extraversion is not measured or observed, nor are we necessarily interested in extraversion, but a pattern of behavior emerges that may be the result of a latent variable, such as extraversion for example. The LCA is a statistical method to identify patterns that result from unobserved underlying common denominators between people. The patterns (latent classes) that emerge from the data can (depending on the needs of the police organization) be interpreted or labeled. For example, the selection committee may wish to categorize the police candidates based on their communication behaviors (observations) into different types of communicators (latent classes) such as extravert communicators, introvert communicators, and nervous communicators.

Table 1. Example of results from behavioral observation with pre-designed behavioral indicators. If a candidate has displayed one of the pre-designed behaviors, it is marked with a 1 in the table. If the behavior is not shown, it is marked with a 0.

Police candidate	Makes eye-contact	Asks questions	Attentive body posture	Listens carefully	Rushes the conversation	Relaxed, open stance	Speaks restlessly
1	0	0	0	0	1	0	1
2	1	0	0	1	1	1	0
3	0	0	1	1	0	0	1
4	0	0	1	1	1	1	1
5	1	1	1	1	1	1	0
6	1	1	1	1	1	1	0

2 The LCA analysis: helpful resources

Conducting an LCA must be done thoroughly and accurately. If expertise is lacking at police organizations, they should consult external experts for this and/or could consult various types of software with accompanying educational resources that are on the market to perform the analysis. In this section, we provide an overview of the software available together with software-specific manuals and general guidelines to conduct an LCA.

2.1 Resources to learn how to conduct LCA

- McCutcheon, A. C. (1987). Latent class analysis. Beverly Hills, CA: Sage.
- Hagenaars, J. A., & McCutcheon, A. L. (Eds.). (2002). *Applied latent class analysis*. Cambridge University Press.

- Vermunt, J. K., & Magidson, J. (2004). Latent class analysis. *The sage encyclopedia of social sciences research methods*, *2*, 549-553.
- Schreiber JB, Latent Class Analysis: An example for reporting results, Research in Social and Administrative Pharmacy (2016), http://dx.doi.org/10.1016/j.sapharm.2016.11.011

2.2 Software to conduct LCA

Commercial
Latent GOLD

- J.K. Vermunt and J. Magidson (2005) Latent GOLD 4.0 User's Guide. Belmont, Massachusetts: Statistical Innovations Inc." https://www.statisticalinnovations.com/wp-content/uploads/LGusersguide.pdf

Mplus

- Muthén, L.K. and Muthén, B.O. (1998-2017). Mplus User's Guide. Eighth Edition. Los Angeles, CA: Muthén & Muthén. https://www.statmodel.com/download/usersguide/MplusUserGuideVer_8.pdf

Gllamm in STATA

- StataCorp. 2019. Stata: Release 16. Statistical Software. College Station, TX: StataCorp LLC. https://www.stata.com/manuals/sem.pdf

PROC LCA in SAS

- Lanza, S. T., Dziak, J. J., Huang, L., Wagner, A. T., & Collins, L. M. (2015). Proc LCA & Proc LTA users' guide (Version 1.3.2). University Park: The Methodology Center, Penn State. https://www.methodology.psu.edu/files/2019/03/proc_lca_lta_1-3-2-1_users_guide-2ggq4d3.pdf

Open source:
R package poLCA

- Linzer, Drew A. and Jeffrey Lewis. 2013. "poLCA: Polytomous Variable Latent Class Analysis." R package version 1.4. http://dlinzer.github.com/poLCA.

3 Results and Interpretation

If we proceed with our example of the police organization looking for strong communicators and suppose they have now conducted a systematic behavioral assessment of a large number of candidates and performed a LCA on the data. Figure 1 shows the results of the LCA with the seven behavioral indicators. To improve accessibility for police organizations to create such LCA output figures, we provide a template in which police organization can enter their own observed behaviors and probabilities scores obtained from the LCA analysis. We refer to the Excel file in the supplementary materials for the figure template. For correct and valuable use of the template, we would like to stress that using probabilities scores derived from a carefully and professionally conducted LCA is required.

The LCA, among other results, renders the following output:

1. The number of latent classes distinguished within the test population that are characterized by specific behavioral patterns.

2. The probabilities that a certain behavior occurs when people belong to a certain latent class. In other words, supposing that a police candidate belongs to a particular class, what is the likelihood ("probability") that the candidate shows the behavior?

Figure 1 shows that the LCA resulted in three latent classes characterized by specific behavioral patterns. If we assume that someone belongs to class 3, then the likelihood (probability) that he or she makes eye contact is .85 (probabilities range from 0 to 1). The likelihood that a police candidate makes eye contact if they belong to class 1 and 2 is lower. As a result, making eye contact is an indicator that someone might belong to class 3. To give another example: If we assume that someone belongs to class 1, then the likelihood (probability) that he or she listens carefully is .80. The likelihood that a police candidate listens carefully if they belong to class 2 and 3 is lower. As a result, listens carefully is an indicator that someone might belong to class 1.

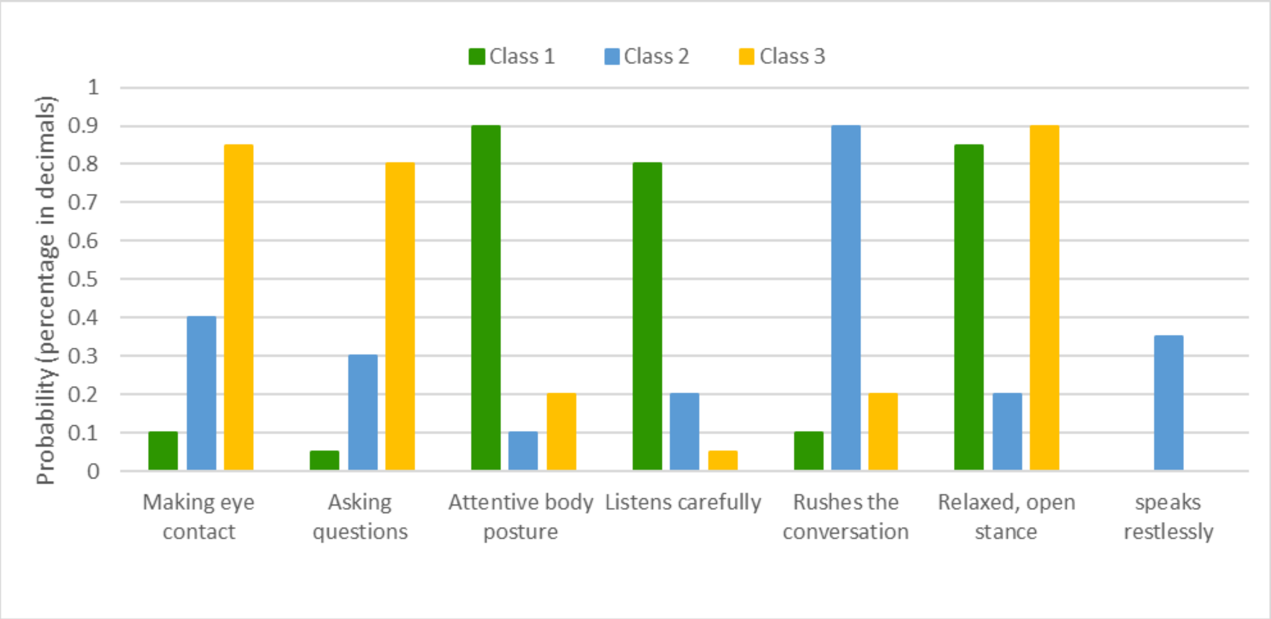

Figure 1. The LCA estimated probabilities of the behavioral indicators occurring in each of the three identified latent classes

The police committee aimed to make a discriminatory distinction in behavioral patterns and distinguish candidates who show behavior associated with good communication skills and candidates who show less good communication skills. The critical question is whether the three classes, with corresponding patterns of behaviors, were related to communication skills or effectiveness. There are two main ways police organizations can figure this out:

- Consult experts within the police organizations, for example, police instructors or experienced police officers, what their own experience is with the (patterns of) behavior and whether these are desirable or problematic regarding communication skills.
- Conduct further analysis to relate behaviors that discriminate between classes to other indicators of communication skills and effectiveness

In this vein, the selection committee may decide to label class 1 as "introvert", class 2 as "nervous," and class 3 as "extravert". Additionally, the police organization may come to the conclusion that both the introvert pattern and the extravert patterns point to strong communicators, albeit in different ways. The nervous pattern points to weak communicators (who either are not selected or may require

extra training when employed). Thus, the introvert communication pattern (class 1) and the extravert communication pattern (class 3) are labeled as 'desirable' classes and the nervous pattern as a 'problematic' class. The behavioral assessment should then give the police organization information on the association between a candidate's displayed behavior and the latent classes.

The strength of the association between observed behavior and a behavioral pattern (i.e., latent class) stems from two things, likelihood and exclusivity. With the likelihood, we mean the probability that behavior occurs for a latent class. With exclusivity, we mean that the probability of that behavior is only high for one latent class and not others. For a strong association between the observed behavior and a behavioral pattern, the behavior must both have a high likelihood and high exclusivity. For instance, Figure 1 shows that both the extravert communication pattern (class 3) and the introvert communication pattern (class 1) have high probabilities for a relaxed, open stance. If a candidate has a relaxed and open stance, this does not inform us whether the candidate is more likely to belong to class 1 or to class 3, as the behavior is probable in both latent classes. To cite another example Figure 1 shows that only the 'nervous' communication pattern (class2) has a probability for speaking restlessly. If a candidate speaks restlessly, this does not inform us whether the candidate is likely to belong to class 2 and not to class 1 or class 3, as the probability that the behavior occurs in the latent class is only low.

To further illustrate these concepts, we take police candidate 3 as an example. This candidate showed an attentive body posture, listened carefully, and spoke restless, but did not make eye contact, did not ask questions, and did not rush the conversation, and did not have a relaxed and open stance. How can we now determine the association between that candidate's behavior and the classes? The fact that police candidate 3 spoke restlessly does not inform us whether the candidate is likely to belong to one of the classes, as the behavior has only a low probability for the nervous communication pattern (class 2). The other two behaviors displayed by candidate 3 give us more insight: only the introvert communication pattern (class 1) has a high probability for an attentive body posture and listen carefully. The extravert communication pattern (class 3) and the nervous communication pattern (class 2) have low probabilities for these behaviors. This suggests a strong association of an attentive body posture and listen carefully with introvert communication pattern (class 1), as these behaviors have a high likelihood and high exclusivity for this latent class. To conclude:
Is candidate 3 one of the extravert communicators? Probably not
Is candidate 3 one of the introvert communicators? Probably yes
Is candidate 3 one of the nervous communicators? Probably not
